# Oxidative Stress and Nonalcoholic Fatty Liver Disease in Hemodialysis Patients

**DOI:** 10.1155/2018/3961748

**Published:** 2018-11-01

**Authors:** Po-Jung Wu, Jin-Bor Chen, Wen-Chin Lee, Hwee-Yeong Ng, Shu-Ching Lien, Pei-Ying Tsai, Chien-Hsing Wu, Chien-Te Lee, Terry Ting-Yu Chiou

**Affiliations:** Division of Nephrology, Department of Internal Medicine, Kaohsiung Chang Gung Memorial Hospital, Chang Gung University College of Medicine and Chung Shan Medical University School of Medicine, Taiwan

## Abstract

**Introduction:**

Nonalcoholic fatty liver disease (NAFLD) is becoming more common around the world and it may progress to cirrhosis and liver failure, increasing mortality risk. In hemodialysis (HD) patients, NAFLD may be a novel risk factor for their high cardiovascular mortality. Heightened oxidative stress is highly prevalent in HD patients. However, the relationship between oxidative stress and NAFLD in HD patients is not well defined.

**Methods:**

We studied seventy-one stable nondiabetic HD patients. Nineteen patients had the diagnosis of NAFLD by ultrasonography. Blood levels of oxidative stress markers were measured in each patient, including thiobarbituric acid reactive substances (TBARS), free thiols, superoxide dismutase (SOD) activities, and glutathione peroxidase (GPx) activity. The copy numbers of mitochondrial DNA (mtDNA) in peripheral leukocytes were also determined. Demographic, biochemistry, and hemogram data were recorded. The two groups of patients were compared in order to determine the factors associated with NAFLD in HD patients.

**Findings:**

Compared to those without NAFLD, nondiabetic HD patients with NAFLD had significantly higher mtDNA copy number and GPx levels. The two groups did not differ significantly in dialysis adequacy, hemoglobin, serum calcium, phosphorus, albumin, liver function tests, or lipid profiles. Regression analysis confirmed mtDNA copy numbers and GPx levels as two independent factors associated with NAFLD. Compared to those with polysulfone, patients dialyzed with cellulose membrane have significantly higher levels of TBARS. However, patients with or without NAFLD did not differ in their use of either dialysis membrane.

**Discussion:**

Oxidative stress (represented by antioxidant defense, GPx) and mitochondrial DNA copy numbers are independently associated with fatty liver disease in nondiabetic HD patients. The diagnostic and therapeutic implications of this key observation warrant further exploration.

## 1. Introduction

Hemodialysis (HD) patients have much higher mortality rate than general population of similar ages [1]. This poor prognosis is, in part, due to high risk for cardiovascular disease and infection [2]. In addition to the traditional risk factors such as diabetes, hypertension, and dyslipidemia, dialysis patients have many nontraditional cardiovascular risk factors, including vascular calcification, oxidative stress, protein-energy wasting, uremic toxins, hyperparathyroidism, and dialysis procedures [3, 4]. Our previous reports showed that nonalcoholic fatty liver disease (NAFLD) is a novel and independent risk factor associated with cardiovascular event in HD patients [5, 6].

NAFLD has emerged as the most common chronic liver disease, affecting 20% to 30% of the worldwide population [7]. It is well recognized that NAFLD is strongly associated with elements of metabolic syndrome, including insulin resistance, obesity, and dyslipidemia. Its pathogenesis involves the complex and dynamic interplays among mediators from liver, adipose tissue, muscle, pancreas, and gut [8]. One of these key mediators is reactive oxygen species (ROS) and oxidative stress, which is defined as an imbalance between the prooxidant molecules and antioxidant defenses. Previous studies have described the associations with oxidative stress and mitochondrial dysfunction in NAFLD patients [9, 10].

Our previous report in HD patients showed that mitochondrial DNA copy number correlates with oxidative stress and predicts mortality [10]. However, the relationships between oxidative stress, mitochondrial DNA copy number, and NAFLD in HD patients have not been described. The purpose of this study is to investigate the associations of oxidative stress, mitochondrial dysfunction, and NAFLD in nondiabetic HD patients. By focusing on nondiabetic patients and reducing the potential confounding influence of insulin resistance, we hypothesized that oxidative stress and mitochondrial dysfunction may contribute to NAFLD in HD patients

## 2. Materials and Methods

### 2.1. Subjects

Seventy-one nondiabetic stable HD patients from the dialysis unit at Chang Gung Memorial Hospital-Kaohsiung Medical Center in Taiwan were enrolled in this study. All of the studied patients received hemodialysis (HD) and none of them received hemodiafiltration (HDF). Inclusion criteria were over 20 years of age and regular 4-hour HD session three times a week for at least 6 months. Exclusion criteria were daily alcohol consumption >20 g, malignancy (within 5 years), acute infection, or hospitalization (within three months). Each participant's medical records were thoroughly reviewed and the demographic, laboratory, and clinical data were collected. Blood samples were obtained before dialysis, as part of the routine clinical protocol. The underlying etiologies of ESRD in these patients include chronic glomerulonephritis (41%), hypertension (32%), polycystic kidney disease (7%), interstitial nephritis (5%), and unknown (15%). Four patients were carriers of hepatitis B, and eight patients hepatitis C; none of them were under active antiviral therapy at the time of this study. This study was approved by the Institutional Review Boards and Ethics Committee in Chang Gung Memorial Hospital, and informed consent was obtained from all participants.

### 2.2. Liver Ultrasonography Evaluation

Gastroenterologists who performed abdominal ultrasonography were blinded to the patients' medical history and blood test results. Nonalcoholic fatty liver disease was defined sonographically based on a comparative assessment of echogenicity relative to the kidneys and in accordance with the proposed criteria by the guidelines of the Asia Pacific Association of Study of Liver Disease [11].

### 2.3. Markers for Oxidative Stress and Mitochondrial Dysfunction

Plasma thiobarbituric acid-reactive substances (TBARS) concentration was measured according to the method described by Ohkawa et al. [12]. Plasma samples were centrifuged and then stored at –80°C until analysis. A standard curve of TBARS was made by serial dilutions of 1,1,3,3-tetraethoxypropane. Results were expressed as micromoles of TBARS per liter (*μ*M). Plasma thiols were measured by direct reaction of the free thiols with 5,5′-dithiobis (2-nitrobenzoic acid) (DTNB), giving 5-thio-2-nitrobenzoic acid (TNB). Thiols levels were calculated from the absorbance using the extinction coefficient of TNB (A412 = 13,600 M−1·cm−1). Superoxide dismutase (SOD) levels were determined by reactions with xanthine oxidase, and absorbance was measured at 440-460nm. As for the glutathione peroxidase activity, reaction with Cumene Hydroperoxide and the absorbance at 340nm were measured and recorded.

The mitochondrial (mt) DNA copy number in peripheral leukocytes was measured by quantitative real-time polymerase chain reaction (PCR) [13] using Roche LightCycler 480r (Roche Applied Science, Mannheim, Germany) apparatus and LightCycler 480 SYBR Green I Master kit (Roche Applied Science, Mannheim, Germany). The DNA from blood samples were extracted using a Gentra DNA Extraction Kit (Qiagen, Germany). To determine the nuclear DNA, the forward primer 5′-GGCTCTGTGAGGGATATAAAGACA-3′ and reverse primer 5′-CAAACCACCCGAGCAACTAATCT-3′ (complementary to the sequences of the chromosome 1 [Chr1] genome loci on 1q24-25) were used to amplify a 97-bp product. For analysis of the mtDNA, we used NADH dehydrogenase subunit 2 (ND2) gene sequences. The forward primer 5′-CACAGAAGCTGCCATCAAGTA-3′ and reverse primer 5′-CCGGAGAGTATATTGTTGAAGAG-3′ were used to amplify an 89-bp product. The PCR reactions were done by initiation at 50°C for 2 minutes, 95°C for 1 minute, 40 cycles at 95°C for 15 seconds, annealing at 60°C for 20 seconds, and extension at 72°C for 15 seconds, and finally holding at 25°C. The values of the threshold cycle number (Ct) of the Chr1 gene and the ND2 gene were determined for each individual quantitative PCR run. The ΔCt [Ct (ND2) - Ct (Chr1)] represents the relative abundance. The quantitative results were expressed as the copy number of mtDNA/cell by 2 × 2-ΔCt. Each measurement was carried out at least 3 times and normalized in each experiment against serial dilutions of a control DNA sample.

### 2.4. Statistical Analysis

Categorical and continuous variables were presented as numbers (proportion/percentage) and mean (±standard deviation [SD]). The differences between the variables of patients with and without NAFLD were tested using the Student t-test. Logistic regression analysis was conducted to examine the associations of oxidative stress markers and other variables with NAFLD. All statistical analyses were conducted using SPSS version 18.0 (SPSS, Inc., Chicago, IL, USA).

## 3. Results

### 3.1. Baseline Characteristics

Among the 71 nondiabetic HD patients, the mean age was 56.0 ± 11.7 years, and the mean hemodialysis vintage was 87.8 ± 63.5 months. Nineteen patients had nonalcoholic fatty liver disease (NAFLD) by ultrasonography, and 52 patients did not (non-NAFLD). At baseline, the two groups had similar age and dialysis vintage (Table 1). Both had good dialysis adequacy (Kt/v and urea reduction ratio [URR]). They also had similar hemoglobin and blood levels of glucose, urea nitrogen, creatinine, and electrolytes. The lipid, iron profiles, and nutrition status (serum albumin) were similar in both groups. Moreover, the two groups had similar liver function tests, including AST, ALT, alkaline phosphatase, and total bilirubin. Finally, there was no significant difference in body mass index.

### 3.2. Nonalcoholic Fatty Liver Disease and Oxidative Stress

Compared to the non-NAFLD, the NAFLD group had significantly higher levels of glutathione peroxidase (GPx) and mitochondrial DNA copy number (Table 1). The two groups did not differ significantly in other oxidative stress markers such as TBARS, thiol, and SOD. Furthermore, regression analysis confirmed mtDNA copy number and GPx levels as two independent factors associated with NAFLD (Table 2).

We also analyzed the associations between oxidative markers and important factors (Table 3). GPx is positively correlated with SOD, but not with TBARS or thiol. MtDNA copy number is positively correlated with TBARS, but not with GPx or SOD. GPx and mtDNA copy number are not correlated. There is a trend for a positive correlation between mtDNA copy number and thiol. SOD is positively correlated with total bilirubin levels. Otherwise, the five oxidative markers did not correlate with liver function tests or lipid profiles.

Relationships between hemodialysis factors, nonalcoholic fatty liver disease and oxidative stress were also examined. Compared to those with polysulfone, patients dialyzed with cellulose membrane have significantly higher levels of TBARS (0.82±0.34 versus 0.64±0.38, P=0.045). The two groups did not differ significantly in other oxidative stress markers examined in this study (data not shown). Patients with or without NAFLD did not differ in their use of either dialysis membrane. They did not differ in their use of vitamin C or activated vitamin D. However, more patients from the non-NAFLD group (29 versus 0%, P=0.008) received intravenous iron supplementation for anemia treatment. None of the studied patients were prescribed with preoral iron supplementation.

Patients with chronic hepatitis B or C have similar prevalence of NAFLD compared to the nonhepatitis subjects. Analysis excluding these 12 patients with hepatitis B or C yielded the same results as above.

## 4. Discussion

Data examining the relationship between oxidative stress and NAFLD in patients with chronic kidney disease is scarce. Our study explored the potential links between oxidative stress and nonalcoholic fatty liver disease in nondiabetic hemodialysis patients. In addition to the markers of oxidative stress and mitochondrial dysfunction, we also incorporated multiple potential factors into our analysis, including BMI, blood glucose, lipid profile, dialysis membrane, and pharmacological agents. Two markers, glutathione peroxidase activity, and mitochondrial DNA copy number stood out as the independent factors associated with NAFLD.

Nonalcoholic fatty liver disease is an increasingly recognized condition that resembles metabolic syndrome in features such as insulin resistance and dyslipidemia [14]. It is characterized by accumulation of lipid in the liver which may lead to oxidative stress and mitochondrial dysfunction [15].

Oxidative stress is an imbalance between oxidant force and antioxidant defense. Precise control of the redox state is essential for cellular homeostasis and structural integrity. This control is achieved by a balance between prooxidant and antioxidant molecules and separation of these molecules into various subcellular compartments. Our study demonstrated that oxidative stress is an important factor associated with NAFLD in HD patients as in other non-CKD populations. Specifically, glutathione peroxidase (antioxidant) activity emerged as a strong independent factor associated with NAFLD. For every 10 U/L increase in GPx, the risk of associated NAFLD is increased by 17%. GPx is an important antioxidant in our body that protects cells from damage by free radicals, such as H_2_O_2_. Samy W. et al. also found that the NAFLD patients (non-CKD) had significantly increased GPx activity, compared to the healthy volunteers [16]. In general, the increased GPx levels may reflect a heightened response to the oxidative stress associated with NAFLD. Moreover, this ability to mount an antioxidant response may also reflect an early stage of liver disease in our HD patients, as suggested by the normal levels of liver function tests. It is possible that as liver injury progresses and elevations in liver function tests become evident, the ability of antioxidant response may be impaired.

We also explored the relationships between oxidants and antioxidants. In our patients, GPx was not correlated with oxidant levels. However, Miguel Roehrs et al. reported that antioxidants (represented by GPx) are correlated with malondialdehyde (MDA, an oxidative stress biomarker) in hemodialysis patients [17]. These divergent results regarding the relationship between oxidative stress and antioxidants may be due to the differences in patient and clinical characteristics. Participants with consumption of vitamin supplementation were excluded from Roehrs' study. By contrast, all of our patients were routinely prescribed with vitamin B complex and folic acid supplementation, which had been shown to alter and disturb the balance between the two countering sides of redox systems [18]. Moreover, some of our studied patients had NAFLD, which was shown to be associated with increased GPx activity in our study.

In our HD patients, additional factors related to uremia may also contribute to the overall oxidative stress. For example, Rossi M. et al. observed an independent association between uremic toxins (indoxyl sulfate and p-cresol) and GPx in patients with chronic kidney disease stages 3-4 [19]. In the in vivo CKD mouse model and in vitro experiments using HepG2 cells, Hamano et al. showed that uremic toxins may induce oxidative stress, leading to alterations in hepcidin regulation and iron metabolism in CKD [20]. It is possible that, in our HD patients, long-term accumulation of uremic toxins may contribute to oxidative stress and NAFLD. In addition to GPx, Samy W. et al. also reported that the NAFLD group (non-CKD) had increased BMI (26 versus 23), lipid profiles, liver function tests (ALT, AST), and blood sugar levels (both fasting and postprandial) [16]. Our study enrolled nondiabetic HD patients so that we can reduce the confounding influence of insulin resistance and focus on the less well-described relationship between oxidative stress and nonalcoholic fatty liver disease. Moreover, our NAFLD and non-NAFLD patients had similar nonoverweight BMI, making obesity a less likely contributing factor to NAFLD in our cohort. Therefore, in contrast to the general population, NAFLD in our studied HD patients is not associated with insulin resistance or obesity but associated with nontraditional risk factors such as oxidative stress and uremic toxins.

Another important independent factor for NAFLD from our study is mitochondrial DNA copy number. Mitochondria perform key cellular functions in fat and energy homeostasis through free fatty acid oxidation, electron transfer, and production of ATP and reactive oxygen species [21]. Disruption of fatty acid oxidation and excessive oxidative stress may be associated with mitochondrial dysfunction, leading to lipid accumulation [22]. Moreover, mitochondria and their DNA are the main targets of free radicals and are susceptible to oxidative damage [23]. Our findings of increased mitochondrial DNA copy numbers in the NAFLD patients and its positive correlations with oxidants (TBARS) may represent a compensatory response to a heightened oxidative injury. Similar to our results, Kamfar S et al. found increased mtDNA copy number associated with NAFLD when comparing 43 Iranian obese NAFLD patients (BMI~44) with 20 obese control subjects (BMI~34) [22]. However, their report showed a much higher increase in mtDNA copy number (3.7-fold, compared to obese control) than our study (58%, compared to non-NAFLD HD patients). On the contrary, two other reports observed a lower mtDNA copy number associated with NAFLD [24, 25]. These differences may be due to obesity and factors related to uremia or dialysis. There are important clinical implications from our findings. Our previous report suggested an association between elevated mtDNA copy numbers and cardiovascular events [5]. Our current study found an association between mtDNA copy numbers and NAFLD. MtDNA copy numbers may serve as an important marker for both cardiovascular and liver injury.

Interestingly, our study found no difference in the levels of other oxidative stress markers (TBARS, thiol, SOD) between NAFLD and non-NAFLD HD patients. Our prooxidant marker, TBARS, may reflect oxidants mainly from lipid peroxidation. However, oxidative stress may also derive from other subcellular sources, including endoplasmic reticulum (ER), lysosomes, peroxisomes, and plasma membrane [26]. It has been estimated that about 25% of the ROS generated in a cell derived from protein oxidation in ER, where a different set of oxidant and antioxidant enzymes may be used. It is possible that ROS from these other cellular organelles were not accounted in our study, leading to an underestimate of the true overall oxidant levels.

Given the lack of DM, obesity, and abnormal liver function tests, it is reasonable to conjecture that NAFLD in our HD patients may be partly related to the underlying uremia and its associated conditions, including dialysis procedure, oxidative stress, metabolic disturbances, and pharmacological modulation [27]. Several groups have described altered antioxidant enzymes and oxidant markers after dialysis [28]. Further research suggests that dialysis membrane affects antioxidant levels, noting higher GPx in patients dialyzed with cellulose acetate (versus polysulfone) membrane [29]. In our study, compared to those with polysulfone, our patients dialyzed with cellulose acetate membrane had higher oxidant (TBARS) levels, but similar levels of other oxidative stress markers. However, patients with different dialysis membrane had similar occurrences of NAFLD. Literature suggests that levels of oxidative markers may also be modulated by other pharmacological agents, including silymarin, vitamin E, and statins [16, 30].

There are several limitations to our study. First, this is a single center study based on retrospective data with a relatively small sample size. Second, this was a cross-sectional observation with measurements of the oxidative and antioxidative biomarkers. Only associations can be determined, but not cause-effect relationships. Serial measurements of the biomarkers over a length of time may provide more sequential information. Third, we did not obtain liver biopsy specimens for the histological assessment of NAFLD. Instead, we used ultrasonography to evaluate fatty liver severity. Ultrasound sensitivity may be compromised when liver contains less than 30% of fat or if BMI is greater than 40 (which are unlikely in our studied patients) [31]. Nevertheless, ultrasonography has the advantage of being noninvasive and inexpensive. Finally, we did not have detailed records of all the potential dietary or pharmacological agents that may affect the prooxidant or antioxidant states. Further studies with a larger sample size will be needed to identify the critical roles of oxidative stress and mitochondrial dysfunction in NAFLD in nondiabetic HD patients.

## 5. Conclusions

Our study showed that nondiabetic and nonoverweight HD patients are still at risk of developing NAFLD, which is independently associated with oxidative stress marker (GPx activity) and mtDNA copy number. Together with the nonassociations with lipid profile levels, these key findings may have several clinical implications: (1) interventions to merely lower lipid profile levels may not prove fruitful; (2) measures to alter or improve the quality (redox status) of lipid profile may worth closer investigation; (3) modulations of uremic toxins or dialysis procedure to improve oxidative stress and NAFLD may warrant future research.

## Figures and Tables

**Table 1:** Comparison of the baseline characteristics of nondiabetic hemodialysis patients with and without nonalcoholic fatty liver disease (NAFLD).

	Non-NAFLD		NAFLD		*P value*
	N=52 (73%)		N=19 (27%)		
Age (years)	56.4	(12.0)	55.0	(10.9)	0.656
Male (%)	44%		32%		0.337
BMI	21.7	(3.8)	20.4	(2.7)	0.164
Hemodialysis vintage (months)	85.6	(65.0)	93.9	(65.0)	0.630
Dialysis membrane (Cellulose %)	38%		58%		0.144
Hepatitis B	3		1		0.907
Hepatitis C	7		1		0.340

**Adequacy of hemodialysis**					
Kt/V	1.51	(0.24)	1.54	(0.29)	0.640
URR	0.77	(0.05)	0.78	(0.07)	0.756

**Hemogram**					
Hemoglobin (g/dL)	10.5	(1.1)	10.5	(0.9)	0.796
Platelet (1000/uL)	194	(41)	201	(41)	0.531

**Biochemistry**					
Sugar (mg/dL)	128	(37)	145	(62)	0.175
Calcium (mg/dL)	9.1	(0.8)	9.1	(0.9)	0.918
Phosphorus (mg/dL)	4.9	(1.3)	4.7	(1.2)	0.402
Intact parathyroid hormone (ug/L)	402	(371)	277	(328)	0.203
Albumin (g/dL)	3.72	(0.30)	3.77	(0.34)	0.513
Blood urea nitrogen (mg/dL)	65	(15)	68	(13)	0.514
Creatinine (mg/dL)	11.6	(2.4)	10.9	(1.6)	0.240
Ferritin (ng/mL)	395	(216)	398	(244)	0.959

**Liver function**					
AST (U/L)	20	(9)	19	(7)	0.658
ALT (U/L)	16	(9)	14	(6)	0.329
Total bilirubin (mg/dL)	0.33	(0.13)	0.29	(0.08)	0.151
Alkaline phosphatase (IU/L)	104	(45)	93	(68)	0.433

**Lipid profile**					
Triglycerides (mg/dL)	156	(86)	174	(96)	0.451
Cholesterol (mg/dL)	185	(33)	194	(42)	0.342
Low-density lipoprotein (mg/dL)	109	(27)	108	(36)	0.961
High-density lipoprotein (mg/dL)	45	(9)	49	(15)	0.150

**Dialysis related medications**					
Vitamin C supplement	6%		0%		0.285
Intravenous iron supplement	29%		0%		0.008
Activated vitamin D3 supplement	21%		21%		0.993

**Oxidative stress biomarkers and mitochondrial function**					
TBARS (uM/L)	0.69	(0.38)	0.80	(0.36)	0.311
Thiol (uM/L)	1.74	(0.40)	1.56	(0.41)	0.111
GPx (U/L)	206.19	(44.66)	233.05	(49.55)	0.033
SOD (U/L)	394.71	(161.38)	455.27	(180.54)	0.179
MtDNA copy number	277.30	(183.40)	439.25	(267.34)	0.006

Values are expressed as mean (SD) or number (%).

*∗P* < 0.05.

*Abbreviations.* SD, standard deviation; NAFLD, nonalcoholic fatty liver disease; URR, urea reduction ratio; AST, Aspartate Aminotransferase; ALT, alanine aminotransferase; BMI, body mass index; TBARS, thiobarbituric acid reactive substances; GPx, glutathione peroxidase; SOD, superoxide dismutase; MtDNA, mitochondrial DNA.

**Table 2:** Multiple logistic regression analysis showing the independent factors associated with nonalcoholic fatty liver disease in HD patients.

	**Odds ratio**	**P-value**
GPx (per 10U/L increase)	1.172	0.037
MtDNA copy number (per 1 copy number increase)	1.039	0.014
BMI (per 1kg/m^2^ increase)	0.880	NS
Sugar (per 1mg/dL increase)	1.017	NS

*∗P* < 0.05.

*Abbreviations*. GPx, glutathione peroxidase; MtDNA, mitochondrial DNA; BMI, body mass index; NS, nonsignificance.

**Table 3:** Correlations between serum oxidative stress markers, mtDNA, and liver profiles.

	TBARS	Thiol	GPx	SOD	MtDNA	AST	ALT	rGT	Alk-P
TBARS	1	0.123	-0.002	-0.130	0.232	0.033	-0.001	0.009	0.053
	(x)	(0.228)	(0.982)	(0.202)	(0.024)	(0.748)	(0.996)	(0.933)	(0.604)

Thiol	0.123	1	-0.017	0.160	0.170	-0.131	-0.077	-0.082	0.114
	(0.228)	(x)	(0.864)	(0.111)	(0.099)	(0.194)	(0.447)	(0.416)	(0.260)

GPx	-0.002	-0.017	1	0.322	0.005	0.007	-0.089	0.012	-0.143
	(0.982)	(0.864)	(x)	(0.001)	(0.959)	(0.947)	(0.379)	(0.904)	(0.157)

SOD	-0.130	0.160	0.322	1	-0.141	-0.085	-0.161	-0.162	-0.065
	(0.202)	(0.111)	(0.001)	(x)	(0.169)	(0.400)	(0.109)	(0.107)	(0.522)

MtDNA	0.232	0.170	0.005	-0.141	1	-0.092	-0.107	-0.127	-0.079
	(0.024)	(0.099)	(0.959)	(0.169)	(x)	(0.374)	(0.298)	(0.216)	(0.442)

Values are expressed as Pearson correlation coefficient (P-value).

*∗P* < 0.05.

*Abbreviations*. TBARS, thiobarbituric acid reactive substances; GPx, glutathione peroxidase; SOD, superoxide dismutase; MtDNA, mitochondrial DNA; AST, Aspartate Aminotransferase; ALT, alanine aminotransferase; rGT, gamma-glutamyltransferase; Alk-P, alkaline phosphatase.

## Data Availability

The data used to support the findings of this study are available from the corresponding author upon request.
